# Commentary: Serum EZH2 is a novel biomarker for bladder cancer diagnosis and prognosis

**DOI:** 10.3389/fonc.2024.1417921

**Published:** 2024-09-24

**Authors:** Tao Wang, Xuan Zhang, Guo-Ming Zhang

**Affiliations:** Department of Laboratory Medicine, Shuyang Hospital, The Affiliated Shuyang Hospital of Xuzhou Medical University, Shuyang, Jiangsu, China

**Keywords:** EZH2, bladder cancer, diagnosis, ROC, biomarker

## 1 Introduction

Feng Li et al. (1) reported that “serum EZH2 is a novel biomarker for bladder cancer diagnosis and prognosis” in an article published in *Frontiers in Oncology*. In this study, to evaluate the diagnostic efficiency of EZH2 for bladder cancer (BC), the investigators collected 115 BC patients and 115 healthy individuals. The ability of EZH2 to discriminate BC patients from healthy individuals was determined via enzyme-linked immunosorbent assay (ELISA), and the diagnostic performance of EZH2 levels was assessed by the area under the curve (AUC) of a receiver operating characteristic (ROC) curve analysis. These researchers found that the EZH2 level was used to distinguish BC patients from healthy individuals, and the AUC was 0.87. Therefore, these authors concluded that EZH2 is a novel diagnostic biomarker for BC diagnosis.

## 2 Commentary and discussion

The study by Feng Li et al. (1) is very interesting as it notes that serum EZH2 is a novel diagnostic biomarker for BC. However, the study has three limitations. First, the number of controls was not more than the number of BC patients, and the controls had only healthy individuals without BC and did not include patients with benign disease. To our knowledge, the number of controls was greater than the number of subjects studied. Therefore, the present study is considered a case-control study (2).

Second, it was neither enrolled in a study cohort consecutively nor was it predesigned for inclusion. The study is a two‐gate design study (2). The BC patients and the controls were from different cohorts. The number of BC patients and the number of controls were not constant or fixed. Therefore, the number of BC patients is crucial, and if the number of BC patients is lower, sensitivity will be underestimated. However, the number was lower for the controls, and there was an underestimation of specificity (2). As such, the sensitivity and specificity of a study were strongly affected by the ratio of researched subjects to controls (3–5). The number of controls (including patients with benign disease and healthy individuals) is crucial for evaluating EZH2 levels for BC diagnosis. The signs, risk factors, and symptoms of healthy individuals are different from those of BC patients. It is not necessary to distinguish in BC and in healthy individuals by using the EZH2 level. A study should use a one-gate design, and it should be based on both exclusion criteria and prespecified inclusion criteria for consecutive enrollment. In this study, BC patients, patients with benign disease, and healthy controls whose EZH2 level should be tested for diagnostic value in BC detection were included.

Third, the authors simply and crudely used *t*-test without testing whether the data had a Gaussian distribution (6). The authors should have described the point selection method for optimal cutoff value, for example, the point corresponding to the maximum Youden index (7) or (and) the points closest to the upper left corner of the ROC curve from the point on the AUC. At the same time, the authors should have also provided 95% confidence intervals of sensitivity and specificity.

In conclusion, EZH2 appears to be a novel diagnostic biomarker for BC. However, researchers should use a one‐gate design for a well‐designed study to avoid the limitations. Healthy individuals and patients with benign disease should be included as controls, and the number of controls should be greater than the number of subjects researched.
